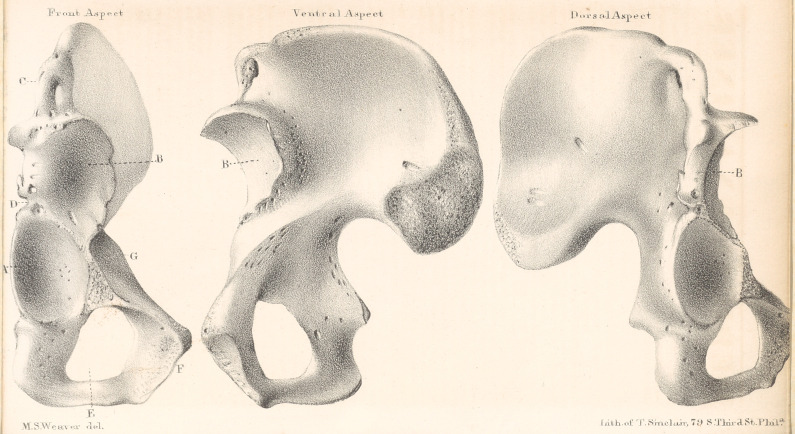# Transactions of the Pathological Society

**Published:** 1840-12-19

**Authors:** Thomas D. Mütter

**Affiliations:** Lecturer on Surgery, &c.


// MEDICAL EXAMINER.

DEVOTED TO MEDICINE, SURGERY, AND THE COLLATERAL SCIENCES.

No. 51.] PHILADELPHIA, SATURDAY, DECEMBER 19, 1840. [Vol. III.

### TRANSACTIONS OF THE PATHOLOGICAL SOCIETY.

*November*, 1840.

### ON LUXATIONS OF THE HIP-JOINT.

By Thomas D. Mütter, M. D., Lecturer on Surgery, &c.

I propose, Mr. President, to direct the attention of the Society, for a few moments only, to certain novel as well as highly interesting facts connected with the displacements of the caput femoris. It is somewhat remarkable, sir, that the luxations of the hip-joint should have remained for so long a period shrouded in the mists of ignorance and uncertainty. Most of the ancient authorities speak of them with great caution, and some pass over the subject entirely; and we find men of almost our own times apparently afraid to trust themselves with their description, and doubting the *possible* occurrence of certain varieties which all of us here must have seen. Thus, Mr. Sharpe, who lived about fifty or sixty years since, is said to have discredited the *possibility* of such a thing as a luxation of the hip-bone. Petit, certainly one of the most acute surgeons of his time, speaks of this class of injuries with much indecision. He declares the displacement upon the dorsum ilii to be *rare*, and wholly discards the luxation *downwards* and *backwards* as impossible.

Benjamin Bell, in his 4th vol., p. 223, thus speaks: "It is said by authors, that the head of the femur may be luxated in various directions, namely, *upward* and *backward*, *upward* and *forward*, *downward* and *backward*, *downward* and *forward*, and I may add *directly downward*. That all of these may happen I cannot take upon me to deny; but I believe few practitioners have met with an instance of the *first* and *third*. The second variety, where the head of the bone passes up upon the os pubis, may happen; as may likewise the *last*, where it is forced *directly down*; but I have never seen any variety except that in which the head of the femur is pushed *downward* and *forward*, and lodged in the *foramen ovale*. All practitioners admit, that the bone is most frequently dislocated in this direction; and an examination of the skeleton, as well as of the recent subject, will show why it should be so." Again he says, at p. 224, "*If ever* this bone be luxated *downward* and *backward*, the leg will be considerably longer," &c. Thus implying a doubt of the possible occurrence of the accident.

Mr. John Bell describes only two luxations of the femur,—that *upwards* upon the dorsum ilii, and that *downwards* in the foramen ovale. (See vol. ii. of his works, edited by Sir Chas. Bell.)

Baron Boyer tells us that luxations of the hip-bone occur in but *four directions*, and that, that *downwards* and *backwards* is *usually*, if not *always* consecutive. From some remarks at page 287 I doubt very much whether he ever met with a case of this variety, and he certainly confounds it with luxation *backwards* and *slightly upwards*, or that in which the head of the bone rests on the upper sciatic notch. (See vol. 4th, page 277.)

Mr. Abernethy, in a very loose chapter upon luxations, speaks of luxation *upwards* and luxation *downwards* of the caput femoris, but alludes to no other of this bone.

Delpech makes but *three* varieties of femoral luxation, and rejects entirely either as *primitive* or *consecutive* that *downwards* and *backwards*.

Sir Astley Cooper, to whom we are indebted for the very best digest of all that is known upon the subject of dislocation, gives us the following division of luxations of the hip:

1st. Upwards, or upon the dorsum of the ilium.

2d. Downwards, or into the foramen ovale.

3d. Backwards, and upwards, or into the ischiatic notch.

4th. Forwards, and upwards, or upon the body of the pubes.

He then goes on to say, "A dislocation *downwards* and *backwards*, has been described by some surgeons who have had opportunities for observation; but I have to remark, that no dislocation of that description has occurred at St. Thomas's or Guy's Hospital, within the last thirty years, or in my private practice; and although I would not deny the possibility of its occurrence, yet I am disposed to believe that some mistake has arisen upon this subject." (See Cooper on Dislocations, Godman's edit., page 58.)

That this high authority is for once at least at fault, is clearly proven by the cases of Mr. Stanley* and Dr. Kirkbride,† which show conclusively that such an accident may occur. Benjamin Bell, it is stated by Phillips,‡ has also reported a case of this injury, but I have been unable to meet with it in the works of this surgeon to which I have had access. Mr.

* See Medico-Chirurgical Trans., vol. xx., paper by B. Travers, jr.

† American Journal of Medical Sciences, No. 31, for 1835, p. 15.

‡ Medical Gazette, No. 10, for 1840.

Keate,* and Prof. Warren, of Boston,† have also reported cases purporting to be luxations of the caput femoris *downwards* and *backwards;* but, as some surgeons have discarded them as such, we will follow their example, as they are not required to prove the existence of the injury in question. The case reported by Ollivier, (Arch. t. iii. p. 545,) as being one of this luxation, is so unsatisfactory in the details, that much caution should be exercised in its reception. There is also another case mentioned by Robert, which, from the signs existing both before and after death, seems to be one of this injury. "The thigh was flexed, adducted, and rotated inwards; elongated to the extent of seven or eight lines; buttock rounded, and very projecting; head of the femur felt just above and behind the *tuberosity* of the ischium. The patient dying, the quadratus was found to be destroyed; the orbicular ligament largely opened at its posterior and inferior portion; the interarticular ruptured." (See Phillips' Lectures, London Medical Gazette, No. x., 1840, p. 646.)

Phillips‡ makes five luxations of the femur, namely, the four usually described by surgeons, and the luxation *directly downwards.*

Liston speaks of only four, not even alluding to those which take place *upwards* and *downwards.* As a further evidence of the looseness with which surgeons treat of these injuries of the hip-joint, I may remark that some of our teachers continue to call that variety in which the head of the bone rests on the greater sciatic notch, the luxation *downwards* and *backwards,* when in reality the head of the femur passes either *directly backwurds,* or *backwards* and slightly *upwards.*

But there yet remains another luxation of the hip, the first case of which is reported by B. Travers, jun., as having occurred in 1832 in the practice of Mr. Green, one of the surgeons to Guy's hospital. Another case of the same accident has been reported by Mr. Morgan, in the Guy's Hospital Reports; and B. Cooper speaks of another as a *partial luxation upwards.* In this variety the head of the bone rests *between the anterior spinous processes of the ilium,* or, *in other words, directly above the acetabulum, instead* of *upon* the *os pubis,* the situation it occupies in luxation *upwards* and *forwards.* Although the case of Mr. Travers is drawn up with but little care, and is wanting in the most valuable portion of a report, the post-mortem appearances, still it is sufficiently interesting for us to bring it before the meeting, and with the permission of the president I will read it.

*Case.*—"Nov., 1832. Edward Mecklam, a sailor, æt. nineteen, states that eight months ago he fell into the hold of a ship, the left buttock striking upon a coil of chain cable. The height might be about twenty feet. The limb, on raising him, was immoveable, everted, and shortened; the difference being considerable. A complete retention of urine followed the accident; but the bladder recovered after the lapse of two days, though the secretion remained turbid for some time.

He did not land in England until four months afterwards, when two attempts were made to reduce the limb without success. He has never suffered from cramp, but the left knee was much swollen after the accident. He describes himself as having met with many severe falls on first attempting to move from his hammock, at the distance of eight weeks from the time of injury. He used to slip up and tumble backwards, with the leg of the affected side under him. The left buttock is flattened; the trochanter is felt rather below and to the outer side of the *anterior* and *superior spinous process* of the ilium. The neck of the bone lies apparently between the *two anterior spinous processes,* so that when the patient is erect, the limb appears slung or suspended from this point. The head of the bone cannot be felt; it is invested by an abundance of bony matter, which extends backwards and inwards over the brim of the pelvis and iliac vessels, occupying in front, nearly the whole space between the inferior spine of the ilium and that of the pubis respectively. There is complete eversion, slight mobility, and imperfect progression with the aid of a crutch."—(See a case of unusual luxation of the thigh bone by B. Travers, Jr., in the xx. vol. of the Med. Chirurgical Trans., p. 112.)

Were this the only case upon record it would not certainly be sufficient to establish the existence of a luxation *directly upwards,* as most of the symptoms might be occasioned by a fracture of the cervix femoris, but the preparation which I have the pleasure to present to the meeting, proves, I think, and clearly too, that Mr. Travers is correct in making "a sixth luxation of the hip bone!"

The bone, of which the specimen I present is a model, is to be found in the valuable collection of the Wistar Museum, and I am indebted to the liberality of Prof. Horner for permission to make this copy. It is a "dissecting room" specimen, and consequently its history is unknown; still, the appearances which it presents clearly prove that it belongs to a class of injuries essentially different from the common luxations of the hip. The location, as well as the aspect of the new socket, moreover, I think, show that it is really an example of a luxation *directly upwards* of the caput femoris. Some who have seen it, suggested the possibility of its having been a case of *spontaneous luxation,* the head of the bone changing its position in consequence of some disease of the socket. Such a view of the case I am not disposed to

* London Medical Gazette, vol. x., p. 19.

† Letter to Hon. Isaac Parker, &c., by I. C. Warren, M. D., Cambridge, 1835.

‡ See Medical Gazette, London, No. 10, for 1840.

receive, inasmuch *as there are no traces of disease* in the *acetabulum, or its immediate vicinity*. The articular cavity is perfectly *smooth*, as is seen in the model, which would not be the case had it been the seat of previous lesion. Nor could the appearances it presents have been the result of a fracture of the cervix or head of the femur. The new socket you perceive is a *smooth, round cup*, such as would form around a hemispherical body, like the head of the femur. Were it a case of fracture, this cavity would be smaller, more irregular, and surrounded by a much larger quantity of callus, while the acetabulum would present marks of the injury, instead of being, as you see, perfectly smooth, and retaining nearly its natural shape and size. The accompanying plate represents very well the peculiar features of the specimen.

A, Acetabulum.
B, New socket.
C, Ant. sup. spine of ilium.
D, Ant. inf. spine of ilium.
E, Foramen ovale.
F, Body of the pubis.
G, Pectineal bos.

The new socket is thus seen to be located *directly above the acetabulum*, between the anterior spinous processes of the ilium, and on the *outside* of the pectineal bos.

In accordance with the above remarks, it would appear, to use the language of Mr. Travers, "that if there be *four lines* of *direction* in *general*, there are *six* particular dislocations of the head of the femur. First, as to *direction*, the displacement may be either *upwards, downwards, inwards*, or *backwards*, (which also signifies outwards.) Second, in addition to the four several luxations so accurately described by Sir A. Cooper, of which *two* occur *internally*, and *two externally*, with reference to the acetabulum as a centre, it must, I think, now be further admitted, that the head of the bone may assume a position *directly above* or *below* the articular cavity. With respect to the dislocations *downwards*, the classification would be much simplified by referring all cases to this variety, where the head of the bone is found to rest *below* the plane of the spinous process of the ischium."

## Figures and Tables

**Figure f1:**